# Supplementary material for: Human sperm steer with second harmonics of the flagellar beat
Source: Nat Commun. 2017 Nov 10;8:1415. doi: 10.1038/s41467-017-01462-y (PMC5680276; doi:10.1038/s41467-017-01462-y)
Supplement: Supplementary file 1 — Supplementary Information [file 41467_2017_1462_MOESM1_ESM.pdf]

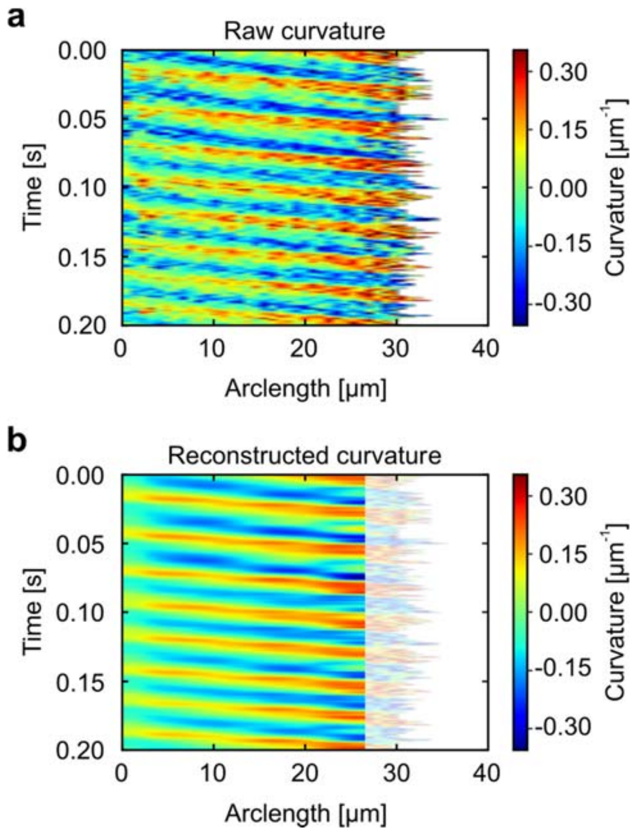

Supplementary Figure 1. Reconstruction of the flagellar curvature. (**a**) Unprocessed curvature of a beating flagellum derived from the tracking points. The number of points is not constant, hence some lines are longer than others. (**b**) The reconstructed curvature from the three most important normal modes of the beat pattern (see Supplementary Fig. 2). The semi-transparent part shows data that has been discarded to have a constant number of flagellar points in time.

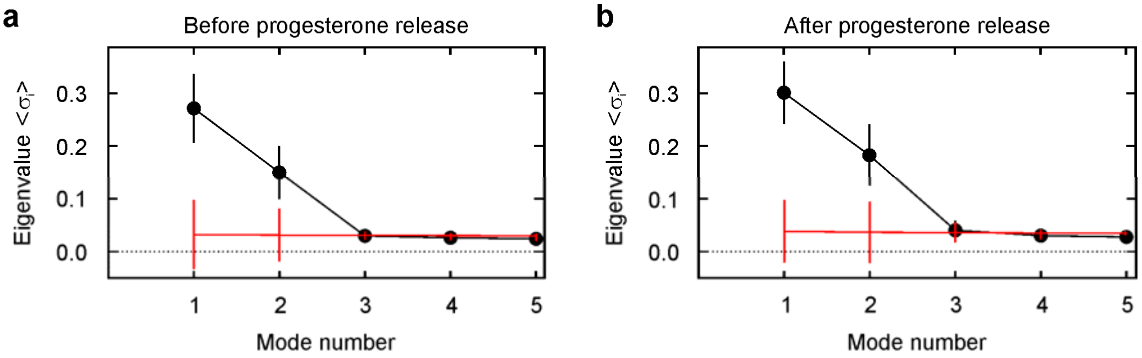

Supplementary Figure 2. Statistically significant beat modes. (**a**) Unstimulated sperm and (**b**) sperm stimulated with progesterone. Comparison of data (black) with random noise (red) shows that only the first three modes are significant. Values are mean ± s.d. (n = 35 panel (a) and n = 26 panel (b)). The third mode is barely above threshold.

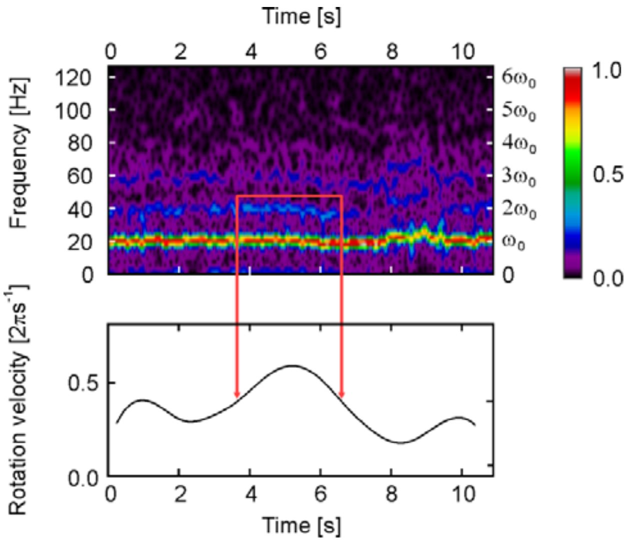

Supplementary Figure 3. Curvature spectrogram. *Upper*: Spectrogram of the curvature at arclength position $s_o \approx 25$ µm from one experiment. The power spectrum is color-coded and normalized to the maximum value. The fundamental mode corresponds to $\omega_o \approx 20$ Hz. Higher harmonics are observed at $2\omega_o$ and $3\omega_o$. Red lines indicate a window of particularly strong second-harmonic intensity. *Lower*: Rotation velocity. Note that the rotation velocity increases with the second-harmonic intensity.

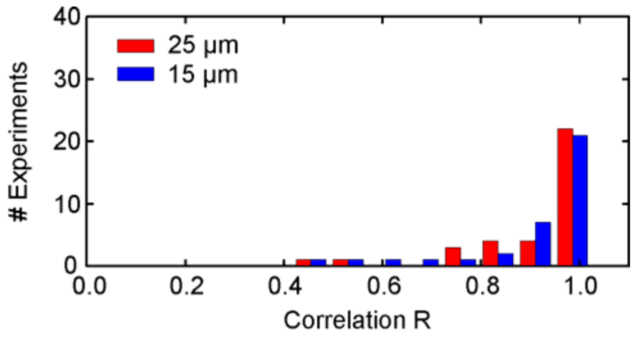

Supplementary Figure 4. Correlation between cell rotation and second-harmonic intensity. Correlation coefficient R of rotation velocity and second-harmonic intensity for unstimulated sperm measured at arclength positions $s_0 \approx 15$ µm and $s_0 \approx 25$ µm.

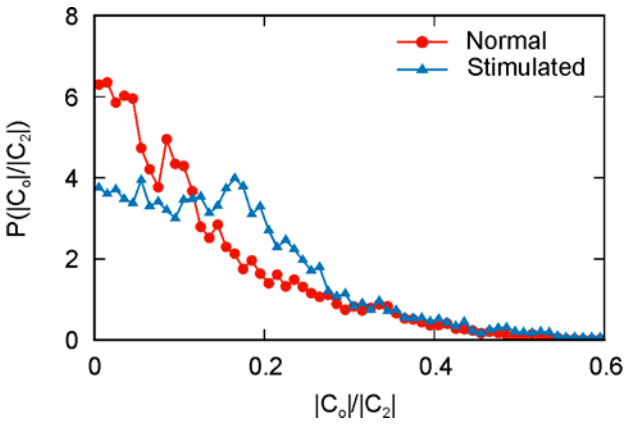

Supplementary Figure 5. Probability density for the ratio of average curvature $|C_0|$ and second-harmonic amplitude $|C_2|$ derived from experiments.

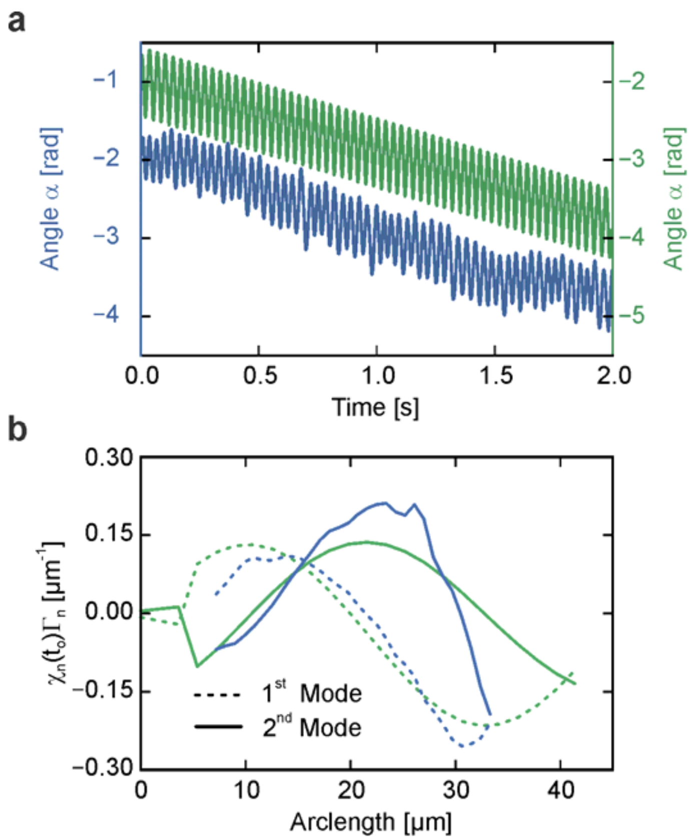

Supplementary Figure 6. Simulated vs. experimental rotation angle and eigenmodes. Comparison between experimental (blue) and simulated (green) rotation angle (**a**) and eigenmodes (**b**). For clarity, t_0 is chosen such that χ_n is maximal. Same simulation as in Fig. 4a in the Main Text and Movie 1. The simulation reproduces fairly well the angular velocity and the curvature eigenmodes.

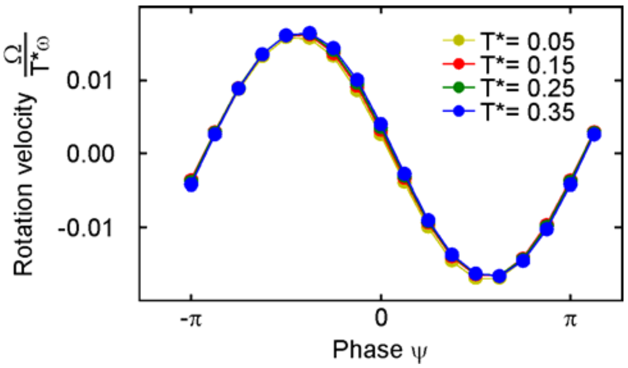

Supplementary Figure 7. Dependency of the simulated rotation velocity with the torque ration and phase. The rotation velocity scales linearly with the applied torque ratio $T^* = T_2/T_1$ and trigonometrically with the torque phase ψ. Parameters are ω = 28 Hz, κ = 2.3 nN µm^2, λ/L = 0.6, and T_1 = 1.38 nN µm.

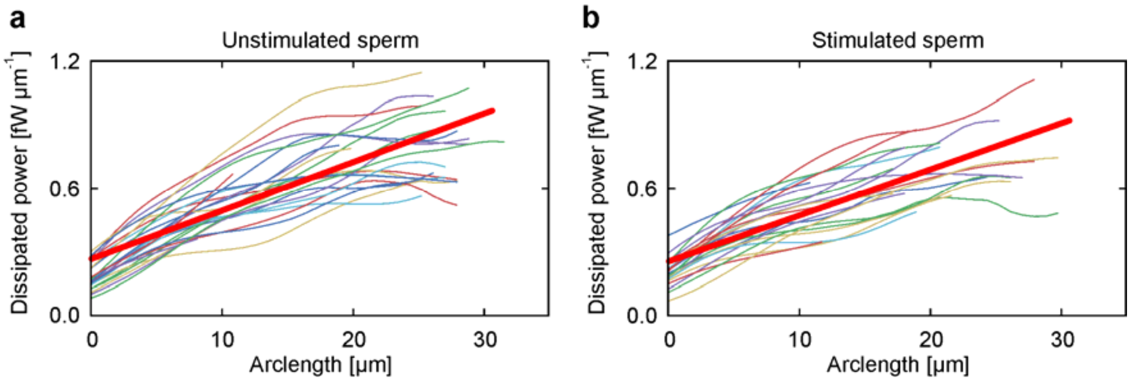

Supplementary Figure 8. Dissipated power. Dissipated power density [fW μm^{-1}] for (**a**) unstimulated and (**b**) stimulated sperm. The ratio between drag coefficients is $\xi_{\perp} / \xi_{\parallel} = 1.81$, with $\xi_{\parallel} = 0.69$ fNs$^{-1}\mu m^{-2}$. The red thick line is the interpolating average using equation (S20).

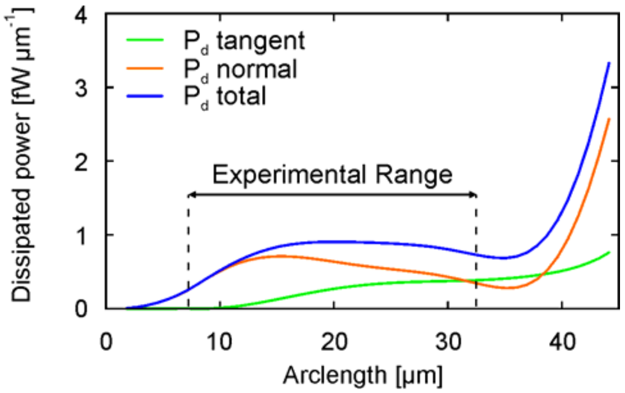

Supplementary Figure 9. Contributions to the total dissipated power P_d by the tangential and normal components of the velocity.

Supplementary Note

Theory of rotation velocity due to the second harmonic

We present here two approaches to derive the rotation velocity generated by the second harmonic. The first approach employs a small-amplitude approximation (equation (2) in the Main Text). This calculation illustrates how the symmetry is broken and produces an average force in the direction perpendicular to the propagation direction. A second approach is based on a small-curvature approximation (equation (4) in the Main Text).

Small-amplitude approximation

For small deviations from a straight line, the flagellum is assumed to be oriented, on average, parallel to the x-axis. The deviation $y(x,t)$ from this line is given by the superposition of two harmonics,

$$y(x,t) = \epsilon\left[y_1 \sin(kx - \omega_o t) + y_2 \sin(kx - 2\omega_o t + \phi)\right] \tag{S1}$$

with $0 < x < L$, $y_2/y_1 \leq 0.3$, and a small expansion parameter ϵ.

The resistive-drag force is

$$\mathbf{f}(x,t) = -\xi_{\parallel}(\mathbf{v}\cdot\mathbf{t})\mathbf{t} - \xi_{\perp}(\mathbf{v}\cdot\mathbf{n})\mathbf{n}, \tag{S2}$$

where the velocity $\mathbf{v}(s,t)$, the tangent $\mathbf{t}(s,t)$, and normal $\mathbf{n}(s,t)$, vectors are given by

$$\mathbf{v}(x,t) = \begin{pmatrix} 0 \\ \partial_t y(x,t) \end{pmatrix},\ \mathbf{t}(s,t) = \frac{1}{N}\begin{pmatrix} 1 \\ \partial_x y(x,t) \end{pmatrix},\ \mathbf{n}(x,t) = \frac{1}{N}\begin{pmatrix} -\partial_x y(x,t) \\ 1 \end{pmatrix}, \tag{S3}$$

with normalization $1/N^2 = 1/(1+(\partial_x y)^2) \approx 1 - (\partial_x y)^2 + O(\epsilon^4)$.

Inserting equation (S3) into equation (S2), we obtain the instantaneous forces acting on the flagellum

$$\begin{aligned} f_x(x,t) &= (\xi_{\perp} - \xi_{\parallel})\partial_t y \partial_x y + O(\epsilon^4), \\ f_y(x,t) &= -\xi_{\perp}\partial_t y + (\xi_{\perp} - \xi_{\parallel})\partial_t y(\partial_x y)^2 + O(\epsilon^5). \end{aligned} \tag{S4}$$

The term $\xi_{\perp}\partial_t y$ in f_y averages out during one period. The average net torque around the tethering point is then computed as

$$T_a = \frac{\omega_o}{2\pi}\int_0^{2\pi/\omega_o} dt \int_0^L dx\, x\, f_y(x,t) = -\epsilon^3 \omega_o y_1^2 y_2 (\xi_{\perp} - \xi_{\parallel}) \cdot \left[kL\sin(kL - \phi) + \cos(kL - \phi) - \cos(\phi)\right]. \tag{S5}$$

In particular, for $\epsilon = 1$ and $2\pi/k \to L$

$$T_a \approx 2\pi\omega_o y_1^2 y_2 \sin(\phi)(\xi_{\perp} - \xi_{\parallel}) \tag{S6}$$

The torque T_a generated by the second harmonic is balanced by the torque generated by the

perpendicular viscous drag T_v. In line with the small-amplitude approximation, we estimate the viscous torque as the torque acting on a straight rod that is tethered at one end and rotates with angular velocity **Ω**,

$$T_v = -\int_0^L dx\, \xi_\perp x(\Omega x) = \frac{\xi_\perp}{3}\Omega L^3. \qquad \text{(S7)}$$

Torque balance ($T_v + T_a = 0$) finally yields

$$\frac{\Omega}{\omega_o} = \frac{\xi_\perp - \xi_\parallel}{\xi_\perp}\frac{6\pi}{L^3} y_1^2 y_2 \sin(\phi). \qquad \text{(S8)}$$

Small-curvature approximation

Second, we consider a description of the flagellar shape in an expansion of small local curvature. This has the advantage that larger perpendicular deviation amplitudes can be included. The flagellar curvature $C(s,t)$ is written as:

$$C(s,t) = \epsilon\left[C_1 \cos(ks - \omega_o t) + C_2 \cos(ks - \omega_o t + \phi)\right], \qquad \text{(S9)}$$

where C_1, and C_2 are the curvature amplitudes of the two harmonics and ϕ is the second-harmonic phase. Given the flagellar curvature $C(s,t)$ at time t, its spatial coordinates $\mathbf{r}(s,t)$ are given by

$$\mathbf{r}(s,t) = \mathbf{r}_o(t) + \int_0^s ds' \begin{pmatrix} \cos\psi(s',t) \\ \sin\psi(s',t) \end{pmatrix}, \qquad \text{(S10)}$$

where $\psi(s,t) = \int_0^s ds' C(s',t) + \psi_o(t)$ is the local angle between the flagellar tangent and the x-axis. Here, we assume that sperm are clamped at their head such that $\psi_o(t) = 0$. By construction, the tangent vector $\mathbf{t}(t,s) = \partial_s \mathbf{r}(s,t)$ is normalized to unity.

The velocity of each line element along the flagellum is

$$\mathbf{v}(t,s) = \partial_t \mathbf{r}(s,t). \qquad \text{(S11)}$$

For convenience, we rewrite the resistive force (equation (S2)) as

$$\mathbf{f}(s,t) = -\xi_\perp \left(1 + \zeta \hat{\mathbf{t}}\hat{\mathbf{t}}^{\mathrm{T}}\right)\mathbf{v}, \qquad \text{(S12)}$$

with $\zeta = \left(\xi_\perp - \xi_\parallel\right)/\xi_\perp$. If a net force is generated, it has to arise from the second term, which is proportional to the friction anisotropy ζ.

The active torque around the tethering point is then obtained (to leading order in ϵ) to be

$$
\begin{aligned}
T_{\mathrm{a}} &= \frac{\omega_{\mathrm{o}}}{2\pi}\int_{0}^{2\pi/\omega_{\mathrm{o}}}\mathrm{d}t\int_{0}^{L}\mathrm{d}s\,\mathbf{r}(s,t)\times\mathbf{f}(s,t) = -\frac{\omega_{\mathrm{o}}\xi_{\perp}\zeta}{2\pi}\int_{0}^{2\pi/\omega_{\mathrm{o}}}\mathrm{d}t\int_{0}^{L}\mathrm{d}s\,\mathbf{r}(s,t)\times\hat{\mathbf{t}}\hat{\mathbf{t}}^{\mathrm{T}}\mathbf{v}(s,t) \\
&= \epsilon^{3}\xi_{\perp}\zeta\omega_{\mathrm{o}}\frac{C_2C_1^2}{8k^6}\left[\left(2k^2L^2+6\right)\sin\left(kL-\phi\right)-kL\cos\left(kL-\phi\right)+6\sin\left(\phi\right)\right].
\end{aligned}
\tag{S13}
$$

In the limit of $\lambda \to L$ and for $\epsilon = 1$, this simplifies to

$$
T_{\mathrm{a}} \approx -\xi_{\perp}\zeta\omega_{\mathrm{o}}C_2C_1^2\pi\frac{4\pi\sin\left(\phi\right)+\cos\left(\phi\right)}{4k^6}. \tag{S14}
$$

Note that the first term in the numerator is usually much larger than the second term. From torque balance and assuming a viscous torque as in equation (S7), we approximate $\Omega = 3T_{\mathrm{a}} / \xi_{\perp}L^3$ by the expression given in equation (4) in the Main Text. Similarly, the average torque generated by an average flagellar curvature C_{o} is obtained

$$
T_{\mathrm{a}} \approx \xi_{\perp}\zeta\omega_{\mathrm{o}}C_{\mathrm{o}}C_1^2\pi\frac{\pi^2-3}{3k^6}. \tag{S15}
$$

Equations (S14) and (S15) together with torque balance demonstrate that the rotation frequency is linear in both the second-harmonic amplitude C_2 and mean curvature C_{o}. Furthermore, for similar values of $|C_{\mathrm{o}}|$ and $|C_2|$ the two terms contribute about equally.

Comparison of second harmonic and average curvature

Supplementary Fig. 5 shows the probability distribution of the ratio $|C_{\mathrm{o}}|/|C_2|$. The second-harmonic contribution is always larger than that of the average curvature; the mean ratio is 0.13 for unstimulated and 0.16 for stimulated sperm. Because the ratio is much smaller than unity, the second harmonic dominates sperm rotation.

Trajectory curvature

The second harmonic generates a rotation of sperm around its tethering point. The rotation velocity Ω depends on the flagellar curvature amplitudes C_1 and C_2, the phase ϕ, and the difference between drag coefficients, $\xi_{\perp}$-$\xi_{\parallel}$. We estimate how these parameters affect the trajectory of freely swimming sperm.

The time needed for sperm to complete a rotation around its center of rotation is $T = 2\pi / \Omega$ where Ω is approximately given by equation (S8) for tethered sperm. Assuming that freely swimming sperm have the same center of rotation, they move during the same time along a trajectory of length vT, with velocity $v = f_{\mathrm{x}} / \xi_{\perp}$. Because $\Omega \ll \omega$, we can ignore the fast wiggling motion due to the beat.

The distance travelled in the time T along a circular trajectory of curvature C_{traj} is

$$2\pi / C_{\mathrm{traj}} = vT = \frac{f_x}{\xi_\perp}\frac{2\pi}{\Omega}, \tag{S16}$$

where $f_x = \frac{\omega_o k}{2}\left(\xi_\perp - \xi_\|\right)\left(y_1^2 + 2y_2^2\right)$. Thus, for $\lambda \rightarrow L$ the trajectory curvature depends on the second-harmonic parameters as $C_{\mathrm{traj}} \approx \frac{y_2}{1+2\left(y_2 / y_1\right)^2}\sin\phi$. Therefore, the second-harmonic amplitude y_2 and phase ϕ determine the swimming trajectory (Fig. 4e in the Main Text and Movie 2).

Sperm model and simulations

The sperm cell is modelled as a single semi-flexible filament, with one end fixed by a stiff harmonic potential. The filament is discretized by beads separated by a distance b between bead centres. Each bead is driven by three types of forces: bending, active, and viscous. The bending force is determined from the bending potential $U_b = \kappa / 2b^3 \sum_{\mathrm{i=0}}^{\mathrm{N-1}} \left(R_{\mathrm{i+1}} - R_{\mathrm{i}}\right)^2$, where R_i is the vector connecting the centres of bead i and i+1, and κ is the bending rigidity, which is obtained by fitting (see below). The active bending torque T is given by equation (5) in the Main Text,

$$T(s,t) = T_1 \sin\left(ks - \omega_o t\right) + T_2 \sin\left(ks - 2\omega_o t - \psi\right). \tag{S17}$$

The viscous forces are modelled by an anisotropic drag. The tangential direction $\hat{t}_i$ at bead i is defined as the line connecting beads i-1 to i+1, and the normal direction is computed by rotating $\hat{t}_i$ counter-clockwise by π/2. At the boundary beads, the tangential direction is identical to the bond direction. To reproduce the mechanics of the beat, the first 7.2 μm of the semi-flexible filament are taken to be inactive, thus mimicking head and midpiece (see Fig.4a in the Main Text). The equations of motion are integrated with an adaptive time-step Velocity-Verlet method.

The parameters were chosen as follows. A bond length b = 1.8 μm is a good compromise between number of points (hence computational performance) and accuracy. This implies that the flagellum is represented by a chain of approximately 25 beads (Supplementary Fig. 6b). The exact number of points depends on the flagellar length in the respective experiment. The

arclength is constrained locally by a harmonic bond potential of stiffness $k = 10$ nN μm^{-1}. We consider a system at low Reynolds number; therefore, the bead mass should not affect the dynamics. Nevertheless, it is useful to assign a small mass (11 pg) to each bead for a stable integration of the equations of motion. The ratio between the perpendicular and the parallel drag coefficients is chosen to be $\xi_{\perp} / \xi_{\parallel} = 1.81$, with $\xi_{\parallel} = 0.69$ fNs μm^{-2}, as measured for bull sperm[1]. The flagellar length, frequency, and wavelength are derived directly from the flagellar waveform. Subsequently, the bending stiffness κ and the torque T_1 are adjusted to minimize the r.m.s. between the experimental and simulated principal modes (Supplementary Fig.6). A second fit adjusts T_2 and ψ to the rotation velocity. The resulting stiffness κ is typically about $\kappa \approx 2$nNμm^2 and thus compatible with known values for sea urchin sperm[2, 3]. Simulation results for beat patterns, rotation velocity, resulting average curvature, and power generation and dissipation are shown in Fig. 4 in the Main Text. In addition, Supplementary Fig. 7 shows that the trigonometric dependence of the rotation velocity Ω with respect to the torque phase ψ agrees well with equation (S8). Note that Supplementary Fig. 7 compares the rotation velocity with respect to the driving torque ratio $T^* = T_2 / T_1$. Thus the simulations support the intuitive idea that T_2 has the same effect as C_2 on the rotation velocity.

Second harmonics of the beat could be produced from the superposition of fundamental and second harmonic waves of active bending torques as described in the Main Text (see Eq. (6)). Alternatively, second harmonics could result from elastic nonlinearities. We tested this hypothesis by using our simulation approach. Specifically, we simulated sperm cells lacking a second harmonic torque, but featuring a constant torque component that results in an average curvature (Supplementary Movie 3). This constant torque has been set such that the resulting average curvature (1) matches that from experiments, or (2) is large enough to produce the cell rotation frequency observed from experiments.

Indeed, including nonlinearities results in a spontaneous second-harmonic frequency. However, when taking an average curvature observed in experiments (case 1), the simulated flagellum does not rotate as fast as tethered sperm. For simulations with either an average curvature set to match experimental rotation frequency (case 2) or a second-harmonic torque, beat shape and flagellar rotation are similar. Nevertheless, a more quantitative analysis reveals that in case (2) the mean amplitude ratio ($\langle C_o/C_2 \rangle \approx 6$) is much larger than that obtained in the second-harmonic-torque simulations ($\langle C_o/C_2 \rangle \approx 0.5$), and is even larger than the experimental ratio ($\langle C_o/C_2 \rangle \approx 0.13$). Thus, we conclude that second-harmonic torques provide the best fit to

the experimental results. It might be possible to consider other forms of nonlinearity. However, to be useful, a detailed understanding of the mechanisms underlying second-harmonic generation is required. The elucidation of the origin of the second harmonic (or even the first one) is beyond the scope of our manuscript.

Generated and dissipated power

The average power density dissipated by the viscous forces at arclength *s* is

$$P_{\mathrm{d}}(s)=\frac{\omega}{2\pi}\int_{0}^{2\pi/\omega}\mathrm{d}t\,\mathbf{f}(s,t)\cdot\mathbf{v}(s,t), \tag{S18}$$

where ***f***(s,t) is the resistive force equation (S2), and ***v***(s,t) is the filament velocity at position *s* and time *t*. Because the torque is the force conjugate to the curvature, the average power generated by the torque is

$$P_{\mathrm{g}}(s)=\frac{\omega}{2\pi}\int_{0}^{2\pi/\omega}\mathrm{d}t\,T(s,t)\partial_{\mathrm{t}}C(s,t). \tag{S19}$$

The torque is given in equation (5) in the Main Text and in equation (S17) Thus, equations (S17), (S18), and (S19), can be used to estimate from experiments and simulations how the power is generated and dissipated along the flagellum.

Experimental results

Supplementary Fig. 8 displays $P_{\mathrm{d}}(s)$ derived from the experimental data. The power dissipation increases approximately linearly towards the tip. For both unstimulated and stimulated sperm, a simple linear fit yields

$$P_{\mathrm{d}}(s)=P_{\mathrm{d}}^{\mathrm{o}}+P_{\mathrm{d}}^{1}s, \tag{S20}$$

where $P_{\mathrm{d}}^{\mathrm{o}}$ = 0.265 ± 0.101 [fW μm^{-1}] and P_{d}^{1} =0.023 ± 0.008 [fW μm^{-2}] for unstimulated sperm and $P_{\mathrm{d}}^{\mathrm{o}}$ = 0.253 ± 0.075 [fW μm^{-1}] and P_{d}^{1} =0.022 ± 0.007 [fW μm^{-2}] after stimulation. Thus, dissipated power does not change upon stimulation.

Simulation results

In contrast to the experiments, the simulations allow a direct calculation and comparison of both the power dissipated in the medium and the power generated by the torques, without any

further assumption and simplification. Figure 4f of the Main Text reveals a quite complex behavior. The generated power P_g displays a maximum roughly 3/4 down the flagellar length, and decreases again towards the tip. In the regime of maximum power consumption, the dissipated power P_d is nearly constant, but then increases sharply towards the tip. This implies that power is transported along the flagellum from the central part of the flagellum toward the tip. Interestingly, Supplementary Fig. 9 shows that in the front part and near the tip, dissipation is mainly due to motion of the flagellum perpendicular to the instantaneous contour, while motion tangential to the contour becomes increasingly important near the tip. These results can be understood qualitatively by considering that the effects of local torques add up to generate large beating amplitudes and velocities in the tip region, while amplitudes and velocities are small near the midpiece due to the tethering constraint.

Discussion

A direct comparison of experimental and simulation results is only possible for the dissipated power. Here, we find that the absolute values and the trends are very similar in the "experimental range" 10-30 μm of arclengths. This is not very surprising given that the beat shapes and beat frequencies from experiments and simulations are very similar (see Fig. 4a in the Main Text and Movie 1), and that the dissipated power is essentially a function of local beat velocities. The strong increase of dissipation near the tip observed in the simulations falls outside the experimental range.

A striking result of the simulations is the pronounced peak of the generated power in the central region of the flagellum. This behavior has important and interesting consequences. In particular, it implies that there is transport of power from the central portion of the flagellum (20 – 35 μm) toward tip and midpiece. Thus, the power is not simply dissipated locally where it is produced. In the case of a swimming sperm, this should be even more pronounced, because part of the power generated by the flagellum is dissipated by the head. Hence, the mechanical structure of the flagellum mediates the power transport to other portions of the sperm cell. Further analysis is needed to gain deeper insights of power generation, thrust, and dissipation, as well as the differences between tethered and freely-swimming sperm.

References

1. Friedrich, B.M., Riedel-Kruse, I.H., Howard, J. & Jülicher, F. High-precision tracking of sperm swimming fine structure provides strong test of resistive force theory. *J. Exp. Biol.* **213**, 1226-1234 (2010).

2. Pelle, D.W., Brokaw, C.J., Lesich, K.A. & Lindemann, C.B. Mechanical properties of the passive sea urchin sperm flagellum. *Cell Motil. Cytoskeleton* **66**, 721-735 (2009).
3. Gadêlha, H., Gaffney, E.A., Smith, D.J. & Kirkman-Brown, J.C. Nonlinear instability in flagellar dynamics: a novel modulation mechanism in sperm migration? *J. R. Soc. Interface* **7**, 1689-1697 (2010).
