# Supplementary material for: Human sperm steer with second harmonics of the flagellar beat
Source: Nat Commun. 2017 Nov 10;8:1415. doi: 10.1038/s41467-017-01462-y (PMC5680276; doi:10.1038/s41467-017-01462-y)
Supplement: Supplementary file 3 — Description of Additional Supplementary Files [file 41467_2017_1462_MOESM3_ESM.pdf]

Description of Additional Supplementary Files

File Name: Supplementary Movie 1
Description: **Beat pattern and sperm rotation.** Experimental recording, corresponding tracking (red), and simulation (blue) which best matches the first two eigenmodes of the curvature and the rotation velocity. The simulated and tracked flagella from previous frames are represented as fading lines (Δt = 2 ms). After the first 210 ms, the movie is accelerated to show the rotation around the tethering point.

File Name: Supplementary Movie 2
Description: **Second-harmonic phase controls swimming direction.** The sperm trajectory in Movie 2 and the corresponding Fig. 4f of the Main Text are generated by slowly and linearly increasing the torque phase ψ of the second harmonic from 0 to 2π. The phase along the trajectory is indicated by colour. For a constant phase ψ, the trajectory is a simple circle.

File Name: Supplementary Movie 3
Description: **Simulations of elastic filaments driven by different active bending torques**. Simulation of three elastic filaments driven by active bending torques including geometric nonlinearities. In orange and black are shown filaments with an active torque oscillating at the fundamental frequency and a constant average torque. The constant torque component results in either an average flagellar curvature (orange) or rotation velocity (black) matching that from experiments. In blue is shown an elastic filament with an active torque including the first and the second harmonic, but no average torque (see Eq. 6 and Supplementary Movie 1).
